# Comparing SARS-CoV-2 Viral Load in Human Saliva to Oropharyngeal Swabs, Nasopharyngeal Swabs, and Sputum: A Systematic Review and Meta-Analysis

**DOI:** 10.1155/2023/5807370

**Published:** 2023-08-10

**Authors:** Mouri R. J. Faruque, Floris J. Bikker, Marja L. Laine

**Affiliations:** ^1^Department of Periodontology, Academic Center for Dentistry Amsterdam, Vrije Universiteit Amsterdam and University of Amsterdam, Amsterdam, Netherlands; ^2^Department of Oral Biochemistry, Academic Center for Dentistry Amsterdam, Vrije Universiteit Amsterdam and University of Amsterdam, Amsterdam, Netherlands

## Abstract

A systematic review and meta-analysis were conducted to investigate the SARS-CoV-2 viral load in human saliva and compared it with the loads in oropharyngeal swabs, nasopharyngeal swabs, and sputum. In addition, the salivary viral loads of symptomatic and asymptomatic COVID-19 patients were compared. Searches were conducted using four electronic databases: PubMed, Embase, Scopus, and Web of Science, for studies published on SARS-CoV-2 loads expressed by *C*_*T*_ values or copies/mL RNA. Three reviewers evaluated the included studies to confirm eligibility and assessed the risk of bias. A total of 37 studies were included. Mean *C*_*T*_ values in saliva ranged from 21.5 to 39.6 and mean copies/mL RNA ranged from 1.91 × 10^1^ to 6.98 × 10^11^. Meta-analysis revealed no significant differences in SARS-CoV-2 load in saliva compared to oropharyngeal swabs, nasopharyngeal swabs, and sputum. In addition, no significant differences were observed in the salivary viral load of symptomatic and asymptomatic COVID-19 patients. We conclude that saliva specimen can be used as an alternative for SARS-CoV-2 detection in oropharyngeal swabs, nasopharyngeal swabs, and sputum.

## 1. Introduction

Coronavirus disease 2019 (COVID-19), caused by SARS-CoV-2 (severe acute respiratory syndrome coronavirus 2), was confirmed as an outbreak reported in Wuhan, China, in December 2019 [1]. Already by March 11th, 2020, it was declared as a global pandemic, indicating the contagiousness and related fast spreading of the virus. By March 16th, 2022, the virus had globally infected over 462 million people with approximately 6 million deaths [2]. To date, these numbers are still increasing.

Most individuals who become infected show mild to moderate flu-like symptoms and recover without hospitalization. Clinical symptoms of COVID-19 are diverse ranging from mild to severe including fever, dry cough, smell- and taste-loss, dyspnea, muscle pain, headache, and respiratory tract infection. In most severe cases, it may lead to lung failure, hospitalization, and death [3]. However, it has been shown that 24% of the population infected with SARS-CoV-2 remained asymptomatic [4, 5]. Several risk factors relate to interindividual differences in sensitivity to COVID-19 including age (fatality rate of patients in the age group 70–80 years is 8% higher than the age groups below [6, 7], gender (higher mortality in males) [8, 9], genetic factors, and underlying comorbidities (cardiovascular diseases, diabetes mellitus, hypertension, chronic kidney disease, and chronic lung diseases) [6]. Differences in viral load kinetics in various body fluids may play a role as well [10–15].

The main human-to-human transmission of SARS-CoV-2 occurs via inhalation of aerosols, generated through coughing, sneezing, or direct contact with mucous membranes of the eyes, mouth, and nose [3, 16–25]. The receptor-binding domain (RBD) of the coronavirus spike (S) glycoprotein, located on the surface of the viral envelope, mediates viral entry into host cells by binding to the ACE2 (angiotensin-converting enzyme 2) receptor. The binding of the S-protein to ACE2 is subsequently primed by a host cell protease, TMPRSS2 (transmembrane protease, serine 2), which facilitates cell entry [20–22]. High expressions of ACE2 and TMPRSS2 are found in the epithelial cells and human acinar granular cells of the salivary glands [22–26]. In line, the salivary glands may serve as a reservoir of the virus facilitating viral replication and shedding of infectious particles into saliva. The viral load profile of SARS-CoV-2 in saliva seems to peak during the first week of symptoms onset [27]. However, the virus may still be detected in low amounts such as approximately ∼2 log10 copies/mL after 20–30 days in saliva, despite the range of salivary antiviral molecules which potentially contribute to counteract the viral load and transmission [1, 13, 14, 27–30].

The collection of respiratory tract secretions such as nasopharyngeal swabs (NPS), oropharyngeal swabs (OPS), and sputum followed by detection of viral genome with RT-PCR has become the gold standard for SARS-CoV-2 screening and diagnosis. However, collection of these matrices has a series of drawbacks regarding discomfort of patients, risk of exposure to healthcare workers, need for specific instruments, and limiting self-collection [31]. In turn, saliva has been regarded to be an attractive matrix for sampling compared to NPS and OPS collection because it offers benefits such as noninvasive and quick and easy sampling with minimum risk of exposure to healthcare workers and decreasing the need of personal protective equipment [11–15, 32–34].

Based on the abovementioned, we hypothesized that SARS-CoV-2 screening and diagnostics in saliva is a good alternative for NPS, OPS, and sputum. It appears, so far, that studies have investigated the detection of SARS-CoV-2 viral loads in saliva specimens indicated in measures of sensitivity and specificity. However, until now, no studies with meta-analysis have compared the SARS-CoV-2 viral load in saliva to other biofluids expressed in *C*_*T*_ values and copies/mL RNA. Therefore, the aim of this systematic review was first to address the SARS-CoV-2 load (expressed in cycle threshold (*C*_*t*_)-value or copies/mL RNA) in human saliva, and secondly, to compare the viral load in saliva with OPS, NPS, and sputum. Furthermore, the SARS-CoV-2 load in saliva samples of symptomatic and asymptomatic COVID-19 patients was compared. A meta-analysis was conducted to systematically compare the viral load data from different studies.

## 2. Materials and Methods

### 2.1. Protocol Registration

This review was registered in PROSPERO International Registration of Systematic Reviews (CRD42021245877) (https://www.crd.york.ac.uk/prospero/display_record.php?RecordID=245877) and written using the Preferred Reporting Items for Systematic Reviews and Meta-Analysis Protocols (PRISMA-P) approach, see Table 1 [35].

### 2.2. Search Strategy and Data Sources

Advanced literature search strategy was applied using four electronic databases including PubMed, Embase, Scopus, and Web of Science. The search strategy was conducted using the combinations of the following key words: (COVID-19 (title/abstract)) OR (coronavirus (title/abstract)) OR (SARS-CoV-2 (title/abstract)) OR (2019-ncov (title/abstract)) AND (saliva (title/abstract)) OR (saliv^*∗*^ (title/abstract)) OR (salivary (title/abstract)) OR (oral (title/abstract)) OR (mouth (title/abstract)) OR (oropharynx (title/abstract)) AND (viral load (title/abstract)). A manual search was conducted in order to include other relevant articles. The search strategy was performed monthly up until April 2021.

### 2.3. Inclusion and Exclusion Criteria

Inclusion criteria included original published scientific articles in English that reported on SARS-CoV-2 load inhuman saliva until April 2021.

Eligibility criteria were conducted using the PICO guidelines [35]:

#### 2.3.1. Population/Patients (P)

Humans, individuals, determined with SARS-CoV-2 load in saliva (all ages).

#### 2.3.2. Intervention/Exposure (I)

SARS-CoV-2 load detected using RT-PCR.

#### 2.3.3. Comparison (C)

SARS-CoV-2 load in OPS and/or NPS and/or sputum, if available.

#### 2.3.4. Outcome (O)

The difference of SARS-CoV-2 load in saliva compared to NPS, OPS, and/or sputum (expressed in *C*_*T*_ values or copies/mL RNA).

Research on the SARS-CoV-2 load was first addressed for saliva. Then, a comparison was made in the viral load in saliva with OPS, NPS, and sputum.

Studies that did not report the viral load in saliva and OPS, NPS, and/or sputum in humans were excluded. Animal studies, reviews, opinion articles, letters to the editor, and case reports were excluded.

### 2.4. Selection Process

One author (MF) performed the initial literature search. Subsequently, three authors (MF, FB, and ML) examined the titles and abstracts of all identified records. Studies were chosen based on the inclusion and exclusion criteria. A single author (MF) extracted the data from the included articles, which again was verified by the authors FB and ML. Disagreements were resolved by discussion.

### 2.5. Data Collection Process

For the included studies, the following parameters were extracted: author(s); year of publication; SARS-CoV-2 viral load in saliva; OPS, NPS, and/or sputum (expressed in *C*_*T*_ value or copies/mL RNA); methods to detect viral load; saliva sampling; total cohort size; percentage of SARS-CoV-2 positive saliva; days of symptom onset; and salivary viral load in symptomatic and asymptomatic COVID-19 patients, if available. If information was missing, corresponding authors were contacted to complete the data.

Firstly, the SARS-CoV-2 load (expressed in *C*_*T*_ value or copies/mL RNA) in saliva was obtained, and secondly, the viral load in saliva was compared to OPS, NPS, or sputum. Finally, the difference in salivary viral load of symptomatic and asymptomatic COVID-19 patients was obtained.

### 2.6. Risk of Bias in Individual Studies

The potential risk of bias in the included studies was assessed using the Quality Assessment Tool for Observational Cohort and Cross-Sectional Studies developed by NIH (National Heart, Lung, and Blood Institute) [36]. Three authors performed the quality assessment independently. Based on the number of “Yes” answers, a rating of good (9–11), fair (5–8), or poor (≤4) was allocated to the individual study. This tool includes 14 questions which were answered by (Yes/No/Not applicable/Not reported/Cannot be determined), see Table 2. Differences in quality rating were discussed by all reviewers (MF, FB, and ML) to reach a consensus.

### 2.7. Data Synthesis

Data on SARS-CoV-2 salivary load were summarized and compared with SARS-CoV-2 load in OPS, NPS, and/or sputum. When ≥3 comparable studies were available, a meta-analysis was conducted using Review Manager (RevMan version 5.4, the Cochrane Collaboration, 2020), where appropriate, the mean (of viral *C*_*T*_ value and viral copies/mL RNA) and standard deviations (SD) were derived. If the mean and SD were not reported, then they were derived from the sample size, median, interquartile range (IQR), and minimum and maximum values using an online calculator at https://www.math.hkbu.edu.hk/~tongt/papers/median2mean.html.Random-effects. A model in RevMan 5.4 was selected to measure the standard mean difference for continuous outcome data with 95% confidence interval (CI). Forest plots were conducted to visualize characteristics of the selected studies; the standard mean difference of viral load in saliva was compared to OPS, NPS, and sputum and the heterogeneity between the studies (*I*^2^). A random effects model was applied for moderate heterogeneity (>30%), otherwise the fixed effects model was applied. The overall mean was obtained. *P* value <0.05 was considered as statistically significant.

## 3. Results

### 3.1. Study Selection

**Figure 1:**
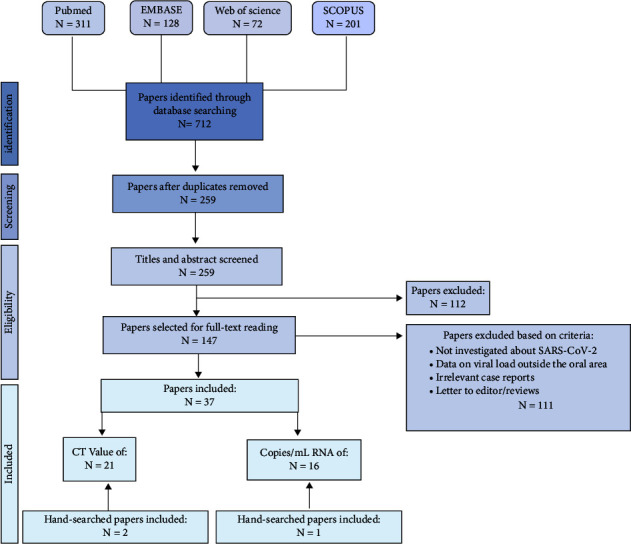
Flowchart diagram (based on PRISMA guidelines) describing the selection procedure of included papers in this systematic review.

A total of 712 articles were retrieved through database search (Figure 1). After duplicate removal, 259 articles were screened by the title and abstract and 147 articles were included for full-text reading after which 111 were excluded. Finally, a total of 37 papers were included. Three additional articles were included by manual search.

### 3.2. Study Characteristics

A total of 21 of the 37 selected studies reported the viral load as a mean or median *C*_*T*_ value (Tables 3–5), while 16 studies reported the viral load in copies/mL RNA (Tables 6–9). Ten articles reported the viral load solely in saliva and 21 articles reported it in saliva compared with OPS, NPS, and/or sputum. The remaining six studies reported the viral load in OPS [1, 49, 50, 59, 60] and sputum combined with saliva [7]. Five of the 31 studies that reported on salivary viral load collected unstimulated whole saliva (UWS) by drooling: the saliva was collected at the bottom of the mouth and then relieved into the collection device [12, 31, 37–39]. Other studies reported saliva collection methods including spitting (three studies) [13, 57, 58], self-collection (eight studies) [11, 14, 33, 42–44, 47, 48], funnel (one study) [32], gargling (one study) [10], saliva stimulated by rubbing outside of the cheeks and then spitting (one study) [15], by coughing (two studies) [41, 54], and by collecting naso-oropharyngeal saliva (two studies) [45, 46]. One study purchased saliva from COVID-19 patients [51]. Seven studies did not report the saliva collection method; however, these studies were included because the viral loads were reported in all cases.

In 24 studies, the viral load dynamics of different respiratory tract samples was evaluated at the early phase of infection (first week), while in five studies, it was assessed in the second week of the infection. The remaining eight studies did not report the days of symptom onset. Furthermore, five studies included the viral load of saliva in symptomatic and asymptomatic COVID-19 patients; in four studies, the mean viral load was reported as *C*_*T*_ value.

### 3.3. SARS-CoV-2 Load in Saliva

The mean SARS-CoV-2 load in saliva derived from 22 studies included 916 patients in total and showed mean *C*_*T*_ values ranging from: 21.5 to 39.6 (Tables 3, 4, 6, and 7). Eleven studies included a total of 216 patients with a mean range of 1.91 × 10^1^ to 5.69 × 10^11^ copies/mL RNA (Tables 6 and 7).

### 3.4. SARS-CoV-2 Load in Saliva Compared with NPS

**Figure 2:**
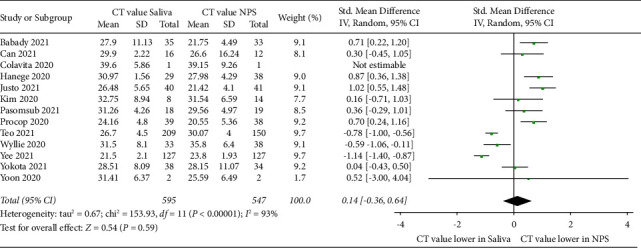
Forest plot showing the standard mean difference of SARS-CoV-2 viral load in *C*_*T*_ values of saliva compared with NPS.

**Figure 3:**
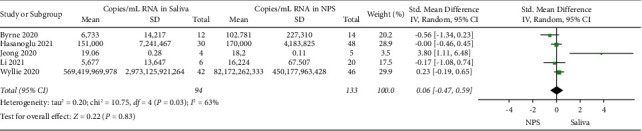
Forest plot showing the standard mean difference of SARS-CoV-2 viral load in copies/mL RNA of saliva compared with NPS.

A total of 13 studies were included for comparison of the standard mean difference in *C*_*T*_ values of saliva and NPS (Figure 2). No significant differences were found in the mean viral load between saliva (overall mean: 26.4) and NPS (overall mean: 26.9 (*P* > 0.05). However, there was considerable heterogeneity between these studies (*P* < 0.00001; *I*^2^ = 93%; 95% CI: −0.36–0.64), demonstrating that these data should be interpreted with caution but might be considered as a trend. Five studies compared the standard mean difference of the viral load given in copies/mL RNA in saliva and NPS (Figure 3). No significant differences were found in the mean viral load between saliva (overall mean: 1.80 × 10^22^) and NPS (overall mean: 2.78 × 10^20^) (*P* > 0.05), and moderate heterogeneity was observed across the studies (*P*=0.03; *I*^2^ = 63%; 95% CI: −0.47–0.59).

### 3.5. SARS-CoV-2 Load in Saliva Compared with OPS

**Figure 4:**
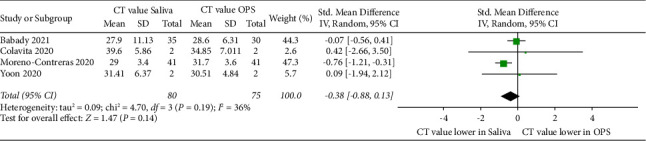
Forest plot showing the standard mean difference of SARS-CoV-2 viral load in CT values of saliva compared with OPS.

Four studies were included for comparison of the standard mean difference in *C*_*T*_ values of saliva and OPS (Figure 4). No significant differences were found in the mean viral load between saliva (overall mean: 28.8) and OPS (overall mean: 30.5) (*P* > 0.05). Moderate heterogeneity was found between the studies (*P*=0.19; *I*^2^ = 36%; 95% CI: −0.88–0.13).

### 3.6. SARS-CoV-2 Load in Saliva Compared with Sputum

**Figure 5:**
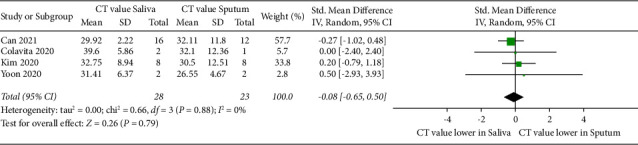
Forest plot showing the standard mean difference of SARS-CoV-2 viral load in *C*_*T*_ values of saliva compared with sputum.

Data from four published studies were selected to compare the mean *C*_*T*_ values of saliva with sputum (Figure 5). No significant differences (*P* > 0.05) and no heterogeneity was found in the mean viral load between saliva (overall mean: 29.3) and sputum (overall mean: 28.8) (*P*=0.88; *I*^2^ = 0%; 95% CI: −0.65–0.50), demonstrating that these data are homogenous.

### 3.7. SARS-CoV-2 Load in Saliva of Symptomatic and Asymptomatic COVID-19 Patients

**Figure 6:**
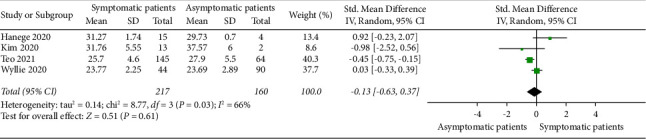
Forest plot showing the standard mean difference of SARS-CoV-2 viral load in *C*_*T*_ value in saliva of asymptomatic and symptomatic COVID-19 patients.

A meta-analysis was conducted to explore the standard mean difference of SARS-CoV-2 load in saliva of symptomatic and asymptomatic COVID-19 patients. Data from four published studies were selected to compare the mean *C*_*T*_ value of saliva in symptomatic and asymptomatic patients (Figure 6). Results indicate that no significant differences were found in the mean viral load between symptomatic (overall mean: 26.06) and asymptomatic patients (overall mean: 25.7) (*P* > 0.05). However, a substantial heterogeneity was obtained between these studies (*P*=0.03; *I*^2^ = 66%; 95% CI: −0.63–0.37).

#### 3.7.1. Risk of Bias Assessment

Overall, 32 studies had a fair risk of bias (Table 2). Three studies were deemed to have a low risk of bias and one study had a high risk of bias. The overall rating in the quality of the studies was fair.

## 4. Discussion

Meta-analysis of 37 included articles revealed that the viral load of SARS-CoV-2 in saliva was comparable to that in NPS, OPS, and/or sputum. Data also disclosed that the viral load in saliva of symptomatic and asymptomatic patients were not significantly different.

Similarities in the viral load of saliva and NPS corresponded to values reported by others [50, 61, 62]. It was shown that saliva has comparable sensitivity to NPS for the detection of SARS-CoV-2 by RT-PCR. Some studies demonstrated higher viral load in saliva compared to NPS [37, 48, 63–65]. In contrast, others showed a lower viral load in saliva; analysis of these values, however, revealed no statistically significant differences [45]. Though, interestingly, it has also been reported that the viral load in saliva peaks earlier, i.e., the first week after infection, and declines less rapidly compared to NPS, suggesting a higher postinfection window of opportunity in saliva for screening and diagnostic purposes [66]. It is thought that the higher viral load and longevity of the virus in saliva may be due to a higher level of ACE2 receptors at various sites in the oral cavity (gingiva, shed epithelial cells in saliva, mucosa, tongue, hard and soft palate, and salivary glands) compared to the nasopharynx [17, 19, 21–25]. Saliva has also been shown to be sensitive enough to detect the majority of viable infections compared to NPS with potential higher likelihood of viral transmission [66].

A considerable heterogeneity was obtained in the meta-analysis of viral load in saliva compared with NPS, which could be explained by the sample size of the studies. To exemplify, the study of Yee et al. (2021) and Teo et al. (2021) had the largest sample sizes: *n* = 127 and *n* = 209, respectively, whereas the sample sizes of other studies varied between 2 and 41. Furthermore, differences in saliva collecting methods may contribute to the heterogeneity. For example, the study of Yee et al. (2021) used a different method for saliva collection compared to the other studies. Furthermore, the authors described that saliva was first stimulated by gently rubbing the outside of the cheeks and subsequently by spitting without interference of coughed-up saliva. Potentially, this method could have stimulated minor salivary glands and parotid glands, secreting predominantly serous saliva potentially loaded with SARS-CoV-2 particles. The saliva sampling methods of the other 11 studies were diverse: six studies reported self-collection [14, 42–44, 47, 48], one study used the so-called drooling method [12], two studies were instructed to collect naso-oropharyngeal saliva [45, 46] and subsequently were asked to spit repeatedly in a sterile cup [45], one study reported coughed-up saliva from the throat [10] while two studies did not report the collection method at all [34, 40]. Currently, there is a lack of a globally accepted and standardized saliva collection protocol for SARS-CoV-2 analysis. However, despite the different saliva collection methods, PCR primers, and conditions, the study set-ups are not likely to have a major influence on the viral loads [67, 68]. The passive drooling technique is generally recommended as standard for saliva collection [69–71]. It is stated that this method provides the greatest sensitivity and allows collecting whole saliva excluding mucous secretions from the oropharynx and sputum [37]. It is an easy and safe technique that can be done with relative simple instructions. As this study revealed that the viral load is comparable in all sample types, we recommend the use of sampling unstimulated saliva, unless other techniques are preferred, e.g., for sake of efficiency, logistic reasons, or standardization. To exemplify, for patients that are intubated and are not able to drool, it is suggested to pipette the saliva sample [70]. Another explanation for the heterogeneity could be that the viral load in saliva changed by food intake and by the circadian rhythm. Wyllie et al. (2020) and Hung et al. (2020) found the highest viral load of 61.5% in the morning, compared to before lunch 23.1%, 3PM, before dinner 7.7%, and at bedtime 0%. Exact times of sampling, however, were not reported. The relative high viral load in the morning may be due to overnight fasting and decreased salivary flow rate during sleep [72]. Consequently, it is, therefore, suggested to refrain from consumption of food and drinks in the morning prior to saliva collection [73]. The same study showed that the salivary flow rate increased after food consumption, which may dilute or wash out the viral RNA [28, 74, 75]. Another factor causing heterogeneity might be the dilution of saliva samples after collection in viral transport medium (VTM). In line, some studies showed that collecting undiluted unstimulated saliva is preferable since the sensitivity and viral detection rates were higher than diluted unstimulated saliva. This processing method also showed no RNA degradation [10, 15, 33]. Most studies were found to have a fair risk of bias, largely due to not providing sample size calculation and power description, as well as not adjusting for potential confounding variables that might impact the outcome such as age and gender.

Meta-analysis from this study is in line with previous studies and demonstrated that no significant differences were found in the viral load of saliva compared to sputum [43, 76–79]. The viral load of sputum showed greater variation than saliva [78, 80, 81]. This could partly be related to the fact that the thick mucus from sputum hampers the viral RNA extraction [82]. It has also been observed that many patients are unable to produce enough sputum and coughs, making it an unsuitable method leading to decreased test sensitivity [77, 83].

We found that the viral load in saliva was comparable to OPS as indicated by *C*_*T*_ values. This finding is in line with other studies [10, 84]. In contrast, however, Moreno-Contreras et al. (2020) found that saliva had a significantly higher viral load compared to OPS, whereas OPS and NPS combined (NPS + OPS) were shown to have a comparable viral load with saliva, suggesting that saliva is a good alternative sampling matrix for NPS + OPS. The reason for the difference between OPS and saliva viral load is unclear, but it is tempting to hypothesize that OPS was not sampled correctly due to the risks associated with this process. A total of 73.1% of NPS positive cases were negative in OPS [85], rendering it a less reliable specimen, as also reported by Khiabani et al. (2021).

Meta-analysis from the current study showed that the mean SARS-CoV-2 loads in saliva of symptomatic and asymptomatic COVID-19 patients are comparable as revealed by *C*_*T*_ values and also shown by other authors [86–88]. Similar viral loads have been also found in other fluids (NPS, OPS, and sputum) [89, 90]. A possible explanation for their comparable viral load could be the shedding of SARS-CoV-2 viral RNA originating from fragmented/degraded genomes of dead viral particles within the oral epithelial cells which has been shed into the saliva of asymptomatic individuals. It has been reported that a high amount of viral RNA does not necessarily mean greater infectivity [89, 91, 92].

It has to be noted that in due course of the current study, new variants of SARS-CoV-2 emerged. Studies on the so-called Omicron variant (B.1.1.529) reported that the viral shedding rate is higher in saliva than in nasal samples [93–95]. It is shown that the salivary Omicron load peaks 1-2 days earlier than the nasal swabs detected by RT-PCR [93]. Marais et al. also concluded that saliva swabs performed better than midturbinate samples up to day 5 postinfection with positive percent agreement (PPA) of 96%. Individuals in the cohort study from Adamson et al. showed to develop symptoms within 2 days after first positive saliva PCR test [93]. Even more, faster and more efficient infection rates have been found for the Omicron variant in the human bronchus compared to the previous SARS-CoV-2 variant, leading to symptoms such as loss of smell and taste which are, therefore, better detected in saliva compared to NPS [93, 94, 96]. Saliva antigen tests and RT-PCR, however, showed a declined sensitivity in Omicron infections after day 5 postinfection with an overall PPA (of RT-PCR) of 96% to approximately 50% [95]. Several studies conclude that saliva swabs are a promising alternative to NPS and midturbinate samples, especially early in infection [93–95]. It is, therefore, advised to use saliva samples as a diagnostic matrix for detecting the Omicron variant, instead of the currently used NPS. Many previous studies have also shown that the diagnostic performance of saliva tests has been successful in other viral infections, i.e., HIV [97–99]. More research is needed to reveal the diagnostic accuracy of saliva, especially in late-stage of infection, for identifying the Omicron and possibly future variants of concern.

## 5. Limitations

Some data of the viral load (in *C*_*T*_ values or copies/mL RNA), SD, and/or IQR were not available and, therefore, could not be included in the meta-analysis. Secondly, the fact that only four studies reported the *C*_*T*_ value and SD of saliva from symptomatic and asymptomatic patients, provided only a small basis for comparison. Thirdly, in some studies, the methods of saliva collection were not reported in detail or at all. Also, saliva characteristics such as viscosity may have influenced the SARS-CoV-2 detection. UWS has usually a mucous consistency, whereas stimulated saliva is relatively serous produced [100].

## 6. Conclusion

This systematic review revealed that SARS-CoV-2 load in saliva is comparable to OPS, NPS, and sputum. Saliva specimen can therefore be used as alternative for SARS-CoV-2 detection since it is noninvasive, convenient, safe, and therefore ideal for mass screening. In addition, it was found that the SARS-CoV-2 loads in saliva of asymptomatic and symptomatic COVID-19 patients were not significantly different.

## Figures and Tables

**Table 1:** PRISMA checklist.

Section/topic	Item #	Checklist item
Administrative information		
Title		
Identification	1a	Identify the report as a protocol of a systematic review
Update	1b	If the protocol is for an update of a previous systematic review, identify as such
Registration	2	If registered, provide the name of the registry (e.g., PROSPERO) and registration number
Authors		
Contact	3a	Provide name, institutional affiliation, and e-mail address of all protocol authors and provide physical mailing address of the corresponding author
Contributions	3b	Describe contributions of protocol authors and identify the guarantor of the review
Amendments	4	If the protocol represents an amendment of a previously completed or published protocol, identify as such and list changes; otherwise, state plan for documenting important protocol amendments
Support		
Sources	5a	Indicate sources of financial or other support for the review
Sponsor	5b	Provide name for the review funder and/or sponsor
Role of sponsor/funder	5c	Describe roles of funder(s), sponsor(s), and/or institution(s), if any, in developing the protocol
Introduction		
Rationale	6	Describe the rationale for the review in the context of what is already known
Objectives	7	Provide an explicit statement of the question(s) the review will address with reference to participants, interventions, comparators, and outcomes (PICO)
Methods		
Eligibility criteria	8	Specify the study characteristics (e.g., PICO, study design, setting, and time frame) and report characteristics (e.g., years considered, language, and publication status) to be used as criteria for eligibility for the review
Information sources	9	Describe all intended information sources (e.g., electronic databases, contact with study authors, trial registers, or other grey literature sources) with planned dates of coverage
Search strategy	10	Present draft of search strategy to be used for at least one electronic database, including planned limits, such that it could be repeated
Study records		
Data management	11a	Describe the mechanism(s) that will be used to manage records and data throughout the review
Selection process	11b	State the process that will be used for selecting studies (e.g., two independent reviewers) through each phase of the review (i.e., screening, eligibility, and inclusion in meta-analysis)
Data collection process	11c	Describe the planned method of extracting data from reports (e.g., piloting forms, done independently, and in duplicate), any processes for obtaining and confirming data from investigators
Data items	12	List and define all variables for which data will be sought (e.g., PICO items and funding sources), any preplanned data assumptions and simplifications
Outcomes and prioritization	13	List and define all outcomes for which data will be sought, including prioritization of main and additional outcomes, with rationale
Risk of bias in individual studies	14	Describe anticipated methods for assessing risk of bias of individual studies, including whether this will be done at the outcome or study level, or both; state how this information will be used in data synthesis
Data		
Synthesis	15a	Describe criteria under which study data will be quantitatively synthesized
	15b	If data are appropriate for quantitative synthesis, describe planned summary measures, methods of handling data, and methods of combining data from studies, including any planned exploration of consistency (e.g., *I*^2^ and Kendall's tau)
	15c	Describe any proposed additional analyses (e.g., sensitivity or subgroup analysis and meta-regression)
	15d	If quantitative synthesis is not appropriate, describe the type of summary planned
Metabias(es)	16	Specify any planned assessment of metabias(es) (e.g., publication bias across studies and selective reporting within studies)
Confidence in cumulative evidence	17	Describe how the strength of the body of evidence will be assessed (e.g., GRADE)

**Table 2:** Studies assessed using the NIH quality assessment tool for observational cohort and cross-sectional studies.

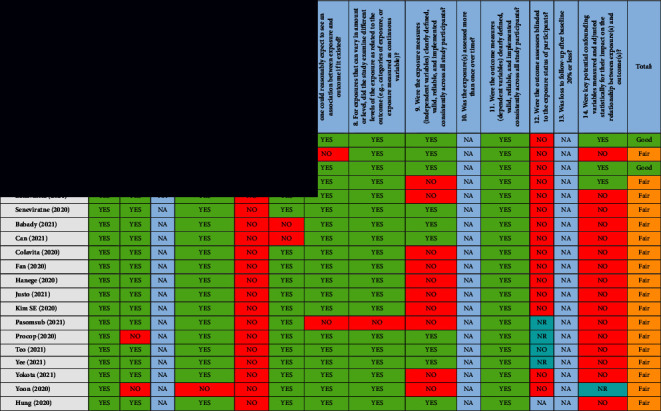
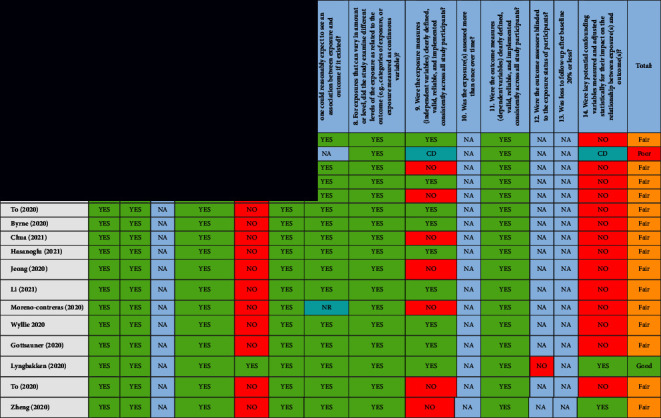

Quality was rated based on the number of “Yes” answers out of 14 questions, a rating of good (9–11), fair (5–8), or poor (≤4). NA = not applicable, NR = not reported, ND = not detected, and CD = cannot be determined.

**Table 3:** Study characteristics of SARS-CoV-2 viral load in saliva indicated by *C*_*T*_ values.

Author	Reference	Study design	Viral load (*C*_*T*_ value) in saliva	SD and IQR	Method to detect viral load	Saliva sample source	% SARS-CoV-2 positive	Total cohort size	Days onset	Symptomatic	Asymptomatic
Azzi (2020)	[37]	Cross-sectional	Mean: 27.16	SD: 3.07	RT-PCR	Drooling (excludes mucous secretions from oropharynx and lower respiratory tracts)	100%	25	0–4	NR	NR

Barat (2021)	[31]	Cross-sectional	Median: 31	IQR: 29–37	RT-PCR	Drooling without restriction on timing or intake of food	6.5%	459	NR	NR	NR
			Mean: 32.4	SD: 6.2							

Basso (2021)	[11]	Prospective cohort	Median: 28.6	IQR: 23.4–32.9	rRT-PCR	Self-collected by the Salivette device (SARSTEDT AG and co, Nümbrecht, Germany), the cotton swab being chewed for at least one minute to stimulate salivation	52.8% (in-patients)	138 (in-patients)	0–7	NR	NR
			Mean: 28.3	SD: 7.2			4.2% (outpatients)	96 (outpatients)			

^*∗*^Bordi (2020)	[38]	Prospective cohort	Median: 32.3	IQR: 11–45	RT-PCR	Drooling, at least 30 min after drinking or eating or washing teeth	53%	164	0–100	*n* = 12	*n* = 14
			Mean: 29.3	SD: 25.6							

Echavarria (2021)	[33]	Prospective cohort	Median: 26.1	IQR: 22.75–30.06	RT-PCR	Self-collection in plastic sterile container	35.1%	174	2	NR	NR
			Mean: 26.3	SD: 7.4							

Seneviratne (2020)	[39]	Randomized control trial	Mean: 27.7	SD: 4.8	RT-PCR	Drooling, refrain from eating, drinking, or performing oral hygiene procedures for at least 30 min	44.4%	36	0–2	NR	NR

All median values, if present, are original and obtained from the publication. ^*∗*^Authors were contacted for the original dataset. NR = not reported, ND = not detected, SD = standard deviation, and IQR = interquartile range.

**Table 4:** Study characteristics of SARS-CoV-2 viral load in saliva compared with OPS, NPS, and sputum indicated by *C*_*T*_ value.

Author	Reference	Study design	Viral load (*C*_*T*_value)	SD and IQR	Method to detect viral load	Saliva sample source	% SARS-CoV-2 positive	Total cohort size	Days onset	Symptomatic	Asymptomatic
Babady (2021)	[10]	Cross-sectional	Saliva mean and median: 27.9	SD saliva: 11.1	RT-PCR	Bringing up saliva from the back of the throat and gargling	Saliva: 12.3% OPS: 10.5% NPS: 11.6%	285	NR	NR	NR
			OPS mean: 28.6 median: 28.9	IQR saliva: 22.2–36.6							
			NPS mean: 21.75 and median: 22.6	SD OPS: 6.3							
				IQR OPS: 24.2–32.3							
				SD NPS: 4.5							
				IQR NPS: 18.5–24.3							

Can (2021)	[12]	Retrospective cohort	Saliva median: 29.89; mean: 29.9	IQR: 28.54–31.27	RT-PCR	Drooling	Saliva: 0.33%	4812	0–12	NR	NR
			Sputum median: 30; mean: 32.1	SD: 2.2			Sputum: 0.25%				
			Oronasopharynx: median: 25.5; mean: 26.6	IQR: 25.85–39.92							
				SD: 11.8			Oronasopharynx: 0.25%				
				IQR: 17.37–36.74							
				SD: 16.2							

Colavita (2020)	[40]	Case report	Saliva mean: 39.6	SD saliva: 5.86	RT-PCR	NR	Saliva: 100%	2	0–30	NR	NR
			NPS mean: 39.15	SD NPS: 9.26			NPS: 100%				
			Throat mean: 34.85	SD throat swab: 7.01			Throat: 100%				
			Sputum: 32.1	SD sputum: 12.36			Sputum: 50%				

^*∗*^Fan (2020)	[41]	Cross-sectional	Hock-a-loogie saliva median: 30; sputum median: 31	IQR NR	RT-PCR	Deep cough 3–5 times and then spitting hock-a-loogie saliva	88.90%	42	8	NR	NR
			Throat: median: 35	SD NR							
			Mean of samples: NR								

^*∗*^Hanege (2020)	[34]	Cross-sectional	Saliva mean: 30.97	SD saliva: 1.56	RT-PCR	NR	Saliva: 76.3% NPS: 100%	38	NR	Saliva *C*_*T*_ value mean: 31.27	Saliva *C*_*T*_ value mean: 29.73
			NPS mean: 27.98	SD NPS: 4.29						SD: 1.74 *n* = 15	SD: 0.7 *n* = 4

Justo (2021)	[42]	Retrospective cohort	Saliva mean: 26.48	SD saliva: 5.65	RT-PCR	Self-collection in sterile tube, avoiding mucous secretions from the oropharynx and sputum	Saliva: 52.63%	76	1–9	*n* = 76 viral load: NR	ND
			NPS mean: 21.42	SD NPS: 4.10			NPS: 54%				

^*∗*^Kim (2020)	[43]	Cross-sectional	Saliva median: 32 mean: 32.8	IQR saliva: 28–38	rRT-PCR	Self-collection using spectrum solutions LLC SDNA-1000 saliva collection device	Saliva: 53% NPS: 93%	15	1–11	Saliva *C*_*T*_ value mean: 31.76	Saliva *C*_*T*_ value mean 37.57
			NP/OP median: 33; mean: 31.5	SD saliva: 8.9			Sputum: 53%			SD: 5.55 *n* = 13	SD: 6 *n* = 2
			Sputum median: 29	IQR NP/OP: 27–35							
			Mean: 30.5	SD NP/OP: 6.6							
				IQR sputum: 24–38							
				SD sputum: 12.5							

Pasomsub (2021)	[44]	Cross-sectional	Saliva median ORF1ab: 32.7; mean: 32	IQR orf1ab saliva: 28.5–35	RT-PCR	Self-collection in sputum collection container (void of coughing)	Saliva: 9% NPS/throat: 9.5%	200	2–11	NR	NR
			median *N* gene: 31.8	SD: 5.2							
			Mean: 31.3	IQR *N* gene saliva: 28.4–33.7							
			NPS/throat median ORF1ab: 32 mean: 31.2	SD: 4.3 IQR ORF1ab NPS/throat: 27.4–34.3							
			median *N* gene: 30.5	SD: 5.5							
			Mean: 29.6	IQR *N* gene NPS/throat: 26.1–32.3							
				SD: 5							

Procop (2020)	[45]	Cross-sectional	Saliva mean: 24.16	SD saliva: 4.80	RT-PCR	Saliva collected via spitting, via nose (by “snuffing” or “snorting” to pull nasal secretion into mouth) and via coughing to produce phlegm or secretions	Saliva: 18.06%	216	NR	*n* = 216 viral load: NR	ND
			NPS mean: 20.55	SD NPS: 5.36			NPS: 17.6%				

Teo (2021)	[46]	Prospective cohort	Saliva tests (*n* = 209) mean: 26.7	SD saliva: 4.5	RT-PCR	Tilt head back, clear the throat and nose, and spit the saliva into the collection bottle. The steps were repeated until the required volume (2 mL) was achieved	Saliva: 62%	200	0–33	Saliva tests (*n* = 145)	Saliva tests (*n* = 64)
			NPS tests (*n* = 150) mean: 30.07	SD NPS: 4			NPS: 44.5%	(337 sets of tests)		*C*_*T*_ value mean: 25.7	*C*_*T*_ value mean: 28.4
										SD: 4.6	SD: 4.7
										Patients (*n* = 88)	Patients (*n* = 112)

^*∗*^Yee (2021)	[15]	Prospective cohort	Saliva samples (*n* = 127) mean: 21.5	Range saliva: 17.9–26.3	qRT-PCR	Gently rubbing the outside of the cheeks and spitting without coughing or clearing throats	Saliva: 26.33%	300	0–43	*n* = 30	*n* = 30
			NPS samples (*n* = 127) mean: 23.8	SD: 2.1			NPS: 29%			Viral load: NR	Viral load: NR
				Range NPS: 21.3–29.0							
				SD: 1.93							

Yokota (2021)	[47]	Prospective cohort	Saliva median: 28.9 mean: 28.51	IQR saliva: 23.1–33.6	qRT-PCR	Self-collected	Saliva: 90% NPS: 81%	42	1–12	*n* = 42	ND
			NPS median: 27.4; mean: 28.15	SD saliva: 8.09						Viral load: NR	
				IQR NPS: 21.3–35.6							
				SD NPS: 11.07							

Yoon 2020	[48]	Case report	Saliva mean: 31.41	SD saliva: 6.37	rRT-PCR	Self-collected	Saliva, NPS, OPS, and sputum 100%	2	1–9	*n* = 2	ND
			NPS mean: 25.59	SD NPS: 6.49						Viral load: NR	
			OPS mean: 30.51	SD OPS: 4.84							
			Sputum mean: 26.55	SD sputum: 4.67							

All median values, if present, are original and obtained from the publication. ^*∗*^Authors were contacted for the original dataset. NR = not reported, NA = not applicable, ND = not detected, NPS = nasopharyngeal swab, OPS = oropharyngeal swab, SD = standard deviation, and IQR = interquartile range.

**Table 5:** Study characteristics of SARS-CoV-2 viral load in oropharyngeal fluid combined with saliva indicated by *C*_*T*_ values.

Author	Reference	Study design	Viral load (*C*_*T*_ value)	SD and IQR	Method to detect viral load	Saliva sample source	% SARS-CoV-2 positive	Total cohort size	Days onset	Symptomatic	Asymptomatic
Hung (2020)	[49]	Cross-sectional	Oropharyngeal saliva median: 34.5	IQR: 32.5–41	RT-PCR	Produce saliva coughed up from the posterior oropharynx (clearing the throat)	89%	18	4–30	CT value: 32	CT value: 34.83
			Mean: 36.1	SD: 6.9						SD: 2.21 *n* = 12	SD: 0.42 *n* = 1

Hon-Kwan Chen (2020)	[50]	Cross-sectional	Posterior oropharyngeal saliva median: 29.7 mean: 31.8	IQR posterior saliva: 27.2–37.2	RT-PCR	Spit posterior oropharyngeal saliva by coughing up via clearing the throat	Posterior saliva: 5.2% NPS: 10.3%	58	NR	NR	NR
			NPS median: 26.8 mean: 27	SD: 12.6							
				IQR NPS: 20.7–33.5							
				SD: 12.2							

All median values, if present, are original and obtained from the publication. NR = not reported, ND = not detected, NPS = nasopharyngeal swab, OPS = oropharyngeal swab, SD = standard deviation, and IQR = interquartile range.

**Table 6:** Study characteristics of SARS-CoV-2 viral load in saliva indicated by copies/mL RNA.

Author	Reference	Study design	Viral load in saliva (copies/mL RNA)	SD and IQR	Method to detect viral load	Saliva sample source	% SARS-CoV-2 positive	Total cohort size	Days onset	Symptomatic	Asymptomatic
^*∗*^Barclay (2020)	[51]	Experimental study	1 × 10^4^	SD: 0.07	RT-PCR	Patient-pooled saliva purchased from BioIVT (saliva-1902492)	35	49 diagnostic remnant samples	NA	ND	ND
			CT value: 28.15								

Kim (2020)	[52]	Cross-sectional	Mean log10: 3.98 (9550 copies/mL RNA)	SD: 0.90	RT-PCR	NR	100	7	0–13	NR	NR

^*∗*^Ning (2021)	[53]	Experimental study	5.65 × 10^7^	SD: 2.28 × 10^4^	RT-qPCR and CRISPR-FDS	NR	43	103	1–7	NR	NR

To (2020)	[54]	Cross-sectional	Median: 3.3 × 10^6^	IQR: 9.9 × 10^2^–1.2 × 10^8^	RT-qPCR	Cough out saliva from the throat into a sterile container	91.7	12	0–7	NR	NR
			Mean: 4.5 × 10^7^	SD: 1.02 × 10^8^							

All median values, if present, are original and obtained from the publication. ^*∗*^Authors were contacted for the original dataset. NR = not reported, NA = not applicable, ND = not detected, NPS = nasopharyngeal swab, OPS = oropharyngeal swab, SD = standard deviation, and IQR = interquartile range.

**Table 7:** Study characteristics of SARS-CoV-2 viral load in saliva compared with OPS, NPS, and sputum indicated by copies/mL RNA.

Author	Reference	Study design	Viral load (copies/mL RNA)	SD and IQR	Method to detect viral load	Saliva sample source	% SARS-CoV-2 positive	Total cohort size	Days onset	Symptomatic	Asymptomatic
^*∗*^Byrne(2020)	[32]	Cross-sectional	Saliva median: 1.83 × 10^3^ mean: 6.73 × 10^3^	IQR saliva: 4.64 × 10^1^–1.7 × 10^4^	RT-PCR	Self-collected with funnel	Saliva: 10.9%	110	NR	*n* = 1234	NR
			NT median: 8.52 × 10^3^ mean: 1.03 × 10^5^	SD saliva: 1.42 × 10^4^			NT: 12.7%				
				IQR NT: 5.08 × 10^1^–2.76 × 10^5^							
				SD NT: 2.27 × 10^5^							

^*∗*^Chua (2021)	[13]	Prospective cohort	Children saliva symptomatic (mean log10): 5.8 (6.32 × 10^5^ copies/mL RNA) asymptomatic saliva (mean log10): 4.5 (3.17 × 10^4^ copies/mL RNA)	IQR saliva symptomatic: 4.9–6.7	RT-PCR	Spit	Saliva: 46%	91	0–18	Mean copies/mL RNA: 6.32 × 10^5^	Mean copies/mL RNA: 3.17 × 10^4^
			Sputum symptomatic (mean log10): 6.4 (2.51 × 10^6^ copies/mL RNA)	IQR saliva asymptomatic: 3.6–5.1						IQR: 7.95 × 10^4^–5.02 × 10^6^*n* = 63	IQR 3.99 × 10^3^–1.26 × 10^5^*n* = 28
			Sputum asymptomatic (mean log10): 5.2 (1.59 × 10^5^ copies/mL RNA)	IQR sputum symptomatic: 4.9–8.1							
				IQR sputum asymptomatic: 3.9–6.1							

^*∗*^Han (2020)	[55]	Case report	Saliva (mean log10): 5.5 (3.17 × 10^5^ copies/mL RNA)	NR	RT-PCR	NR	100%	1	6–10	NR	NR
			Neonate OPS: 1.3 × 10^8^								

^*∗*^Hasanoglu (2021)	[56]	Prospective cohort	Saliva (mean log10): 5.18 (1.51 × 10^5^ copies/mL RNA)	Saliva (min–max log10): 2.87–7.47 (7.42×10^2–2.96×10^7 copies/mL RNA)	RT-qPCR	NR	Saliva: 50% NPS: 80%	60	1–14	*n* = 22/45	*n* = 8/15
			Saliva (median log10): 5.25 (1.78 × 10^5^ copies/mL RNA)	SD: 7.2 × 10^6^							
			NPS (mean log10): 5.23 (1.7 × 10^5^ copies/mL RNA)	NPS (min–max): 2.22–7.27 (1.66 × 10^2^–1.86 × 10^7^ copies/mL RNA)							
			NPS (median log10): 5.11 (1.29 × 10^5^ copies/mL RNA)	SD: 4.2 × 10^6^							

^*∗*^Jeong (2020)	[29]	Case report	Saliva (mean log10): 1.28 (19.06 copies/mL RNA)	SD saliva: 0.28	RT-PCR	NR	Saliva: 80%	5	8–30	NR	NR
				SD NPS: 0.11			NPS: 100%				
			NPS (mean log10): 1.26 (18.2 copies/mL RNA)								

Li (2021)	[57]	Prospective cohort	Saliva (copies/test) mean: 5.68 × 10^3^	SD saliva: 1.36 × 10^4^	ddPCR	Spit	Saliva: 16.2%	37	7–69	NR	NR
			NPS (copies/test) mean: 1.62 × 10^4^	SD NPS: 6.75 × 10^4^			NPS: 54.1%				

^*∗*^Moreno-contreras (2020)	[58]	Cross-sectional	Saliva median: 3.5 × 10^10^	IQR NR	RT-qPCR	Spit	Saliva: 37.2%	253	NR	NR	NR
			OPS median: 3.9 × 10^8^	IQR NR			NPS: 31.6%				
			Saliva (CT value mean): 29 (*n* = 41)	SD saliva (CT value): 3.4							
			OPS (CT value mean): 31.7 (*n* = 41)	SD OPS (CT value): 3.6							

^*∗*^Wyllie 2020	[14]	Prospective cohort	Saliva mean: 5.69 × 10^11^	SD saliva: 2.97 × 10^12^	RT-PCR	Self-collected (spit repeatedly in sterile urine cup, excluding bubbles)	Saliva: 29.6% NPS: 32.4%	142	2–34	Saliva copies/mL RNA mean: 5.69 × 10^8^	Saliva copies/mL RNA mean: 6.38 × 10^4^
			NPS mean: 8.22 × 10^10^	SD NPS: 4.5 × 10^11^						SD: 2.97 × 10^9^*n* = 39	SD: 3.8 × 10^4^*n* = 3
			Saliva (CT value mean): 31.5	SD saliva (CT value): 8.1						Saliva CT-value mean: 23.77	Saliva CT-value mean: 23.69
			NPS (CT value mean): 35.8	SD NPS (CT value): 6.4						SD: 2.25 *n* = 44	SD: 2.89 *n* = 90

All median values, if present, are original and obtained from the publication. ^*∗*^Authors were contacted for the original dataset. NR = not reported, ND = not detected, NT = nasal-throat, NPS = nasopharyngeal swab, OPS = oropharyngeal swab, SD = standard deviation, and IQR = interquartile range.

**Table 8:** Study characteristics of SARS-CoV-2 viral load in oropharyngeal fluid combined with saliva indicated by copies/mL RNA.

Author	Reference	Study design	Viral load (copies/mL RNA)	SD and IQR	Method to detect viral load	Saliva sample source	% SARS-CoV-2 positive	Total cohort size	Days onset	Symptomatic	Asymptomatic
Gottsauner (2020)	[59]	Cross-sectional	Mouth and throat median: 1.8 × 10^3^	IQR: 3.1 × 10^2^–4.7 × 10^4^ copies/mL	RT-PCR	Gargle mouth and throat with 20 mL 0.9% NaCl for 30 s	83	12	1–5	NR	NR
			Mean: 1.8 × 10^4^	SD: 4.02 × 10^4^							

Lyngbakken (2020)	[60]	Randomized controlled trial	OPS (mean log10): 4.5 (3.17 × 10^4^ copies/mL RNA)	NR: SD copies/mL RNA	RT-qPCR	Swabs were rotated for ten seconds on posterior oropharyngeal mucosal membrane (over both tonsils, soft palate, and posterior oropharynx)	49	51	2	NR	NR
			OPS (CT value mean): 34.54	SD OPS (CT value): 6.53							

^*∗*^To (2020)	[1]	Observational cohort	Posterior oropharyngeal saliva (median log10): 5.2 (1.59 × 10^5^ copies/mL RNA)	IQR: 4.1–7	RT-qPCR	Coughing by clearing throat and saliva from intubated patients were obtained by endotracheal aspiration	87	23	0–30	NR	NR

All median values, if present, are original and obtained from the publication. ^*∗*^Authors were contacted for the original dataset. NR = not reported, ND = not detected, NPS = nasopharyngeal swab, OPS = oropharyngeal swab, SD = standard deviation, and IQR = interquartile range.

**Table 9:** Study characteristics of SARS-CoV-2 viral load in sputum combined with saliva indicated by copies/mL RNA.

Author	Reference	Study design	Viral load (copies/mL RNA)	SD and IQR	Method to detect viral load	Saliva sample source	% SARS-CoV-2 positive	Total cohort size	Days onset	Symptomatic	Asymptomatic
^*∗*^Zheng (2020)	[7]	Retrospective cohort	Saliva + sputum (median log10): 5	IQR: 4–5.9	qRT-PCR	By coughing out saliva from the throat into a sterile container	100	96	0–55	NR	NR
			(1 × 10^5^ copies/mL RNA)								

All median values, if present, are original and obtained from the publication. ^*∗*^Authors were contacted for the original dataset. NR = not reported, ND = not detected, NPS = nasopharyngeal swab, OPS = oropharyngeal swab, SD = standard deviation, and IQR = interquartile range.

## Data Availability

The data used to support the findings of this study are available within the article. This is a review based on published data.
